# Do Clear Aligners Release Toxic Chemicals?—A Systematic Review

**DOI:** 10.3390/jfb16050173

**Published:** 2025-05-10

**Authors:** Mariana Ferreira, Hélder Costa, Nélio Veiga, Maria J. Correia, Ana T. P. C. Gomes, Pedro C. Lopes

**Affiliations:** 1 Faculty of Dental Medicine, Universidade Católica Portuguesa, 3504-505 Viseu, Portugal; s-maalbaferreira@ucp.pt (M.F.); hecosta@ucp.pt (H.C.); nveiga@ucp.pt (N.V.); mcorreia@ucp.pt (M.J.C.); apgomes@ucp.pt (A.T.P.C.G.); 2 Center for Interdisciplinary Research in Health, Universidade Católica Portuguesa, 3504-505 Viseu, Portugal

**Keywords:** clear aligners, advanced polymer materials, cytotoxicity, orthodontics

## Abstract

Clear aligners are a modern orthodontic solution designed to address dental malocclusions discreetly and effectively. In terms of clinical side effects, the most reported issues among aligner users are mild irritation of the oral mucosa, localized inflammation, and hypersensitivity. The use of advanced polymer materials in clear aligners, such as polyurethane and PET-G, has gained widespread acceptance due to their mechanical properties, biocompatibility, and aesthetic appeal. However, concerns persist regarding their potential to release chemical compounds. Our goal is to understand the impact of clear aligner toxicity on the oral cavity of orthodontic patients. An extensive systematic search was conducted in the electronic databases PubMed, Scopus, and Cochrane to identify articles with relevant data. This systematic review was conducted following the Preferred Reporting Items for Systematic reviews and Meta-analysis guidelines (PRISMA) to answer a question formulated according to the Population, Intervention, Comparison, and Outcomes. Four hundred and thirteen articles potentially relevant were identified and after applying PRISMA guidelines and inclusion/exclusion criteria, seven articles were included in this review. Our results suggest that clear aligners are generally safe, but concerns remain regarding the chemical leaching of thermoplastic materials, bacterial accumulation due to reduced saliva flow, and mild inflammatory responses. Our review emphasizes that although most materials are biocompatible, some exhibit moderate cytotoxicity, with the potential to impact these patients’ oral health, underscoring the need for continued research and improvements in material composition.

## 1. Introduction

The field of orthodontics has seen substantial advancements, particularly with the development of clear aligners, which offer patients a discreet alternative to traditional metal braces. Clear aligners are increasingly popular for treating mild to moderate malocclusions, and they are associated with fewer adverse effects on oral health compared to fixed appliances [1,2]. These aligners are especially appealing to adults who seek effective yet inconspicuous treatments, reflecting a shift in orthodontic approaches that prioritize patient comfort and lifestyle compatibility [1].

Clear aligners, a popular alternative to traditional braces, are made from advanced polymer materials that provide flexibility, durability, and transparency. These medical-grade polymers, such as polyurethane or polyethylene terephthalate glycol (PET-G), are designed to withstand mechanical stress while remaining biocompatible [3,4]. Additionally, gels play a crucial role in this type of orthodontic treatment, often used for teeth whitening or as lubricants to enhance patient comfort. Some aligners incorporate specialized gels infused with fluoride or antimicrobial agents to promote oral health during treatment. For example, products like EverSmile WhiteFoam or OrthoFoam can be applied inside aligners such as Invisalign to help reduce bacteria, prevent cavities, and provide mild whitening effects [4,5]. These types of advanced orthodontic materials are designed with biocompatibility and safety in mind, meeting stringent requirements to ensure they do not induce cytotoxic effects or release harmful substances during use [6,7].

The design and function of clear aligners are rooted in digital orthodontic technology, which begins with a scan or digital impression of the patient’s teeth that is then used to create an accurate 3D model of the dental arches, allowing the orthodontist to develop a comprehensive treatment plan [8]. Using computer-aided design/computer-aided manufacturing (CAD/CAM) technologies, a series of aligners are manufactured to guide the teeth through progressive movements toward ideal alignment.

In terms of effectiveness, research indicates that clear aligners can produce results comparable to traditional fixed braces for certain types of malocclusions. For example, the studies by Miller et al. [9] suggest that aligners are particularly effective for resolving minor crowding and spacing problems, as well as for achieving controlled tipping and limited tooth rotations. However, the limitations of aligners become apparent in more complex orthodontic cases, such as significant root movement or the correction of severe malocclusions, where fixed appliances can still offer superior results [10].

Furthermore, while the benefits of clear aligners are significant, understanding the potential for these materials to degrade over time and the subsequent health risks is critical for patient safety. The degradation of the aligner material could potentially lead to the release of toxic substances that may have harmful effects, ranging from mild irritation to more severe systemic health concerns [11]. Although many aligners are manufactured with BPA-free plastics, there are still concerns about the possibility of leaching other bisphenol A derivatives or additives under certain conditions, such as exposure to high temperatures or acidic environments [12,13]. Katras et al., 2021 have found that several aligners still release BPA although below toxic levels [14]. Furthermore, a study by Ryokawa et al. [15] found that certain orthodontic thermoplastics can elicit inflammatory responses when tested in human gingival fibroblasts. Although these effects were mild and generally below clinically significant levels, they highlight the importance of understanding the cellular interactions between aligners and oral tissues, especially for patients with pre-existing sensitivity [7].

In terms of clinical side effects, the most reported problems are mild irritation of the oral mucosa, localized inflammation, and hypersensitivity. These symptoms are typically attributed to friction between the aligner and gum tissue rather than chemical toxicity but could also be related to individual oral pH levels, diet, and hygiene practices [6,9]. However, in some cases, patients reported experiencing more persistent symptoms, such as pain and swelling in the gums, possibly related to localized inflammatory responses triggered by small chemical releases [16].

In response to these concerns, manufacturers have taken steps to reduce the potential cytotoxicity of aligners. The development of BPA-free materials and the use of alternative plasticizers have contributed to significant improvements in aligner safety [17]. Additionally, some companies are exploring the use of biocompatible coatings to reduce direct contact between the plastic and the oral mucosa, potentially lowering the risk of both chemical leaching and mucosal irritation. These innovations reflect a shift towards safer and more biocompatible aligners, though rigorous testing remains essential to validate the efficacy of these new materials under clinical conditions [1,18]. This systematic review aims to understand the impact of the toxicity of clear aligners on the oral cavity of patients who use clear aligners and consequently understand how the materials that make up the aligners are related to the possible toxicity they cause. With this knowledge, dentists will be able to act correctly and preventively to improve the oral health of these patients. This systematic review stands out for the fact that it only describes information about clear aligners and their side effects. The existing systematic reviews include aligners, retainers, and other types of orthodontic devices which, in addition to acrylic, use other materials such as metals.

To reach the goals, the literature search was conducted following the Preferred Reporting Items for Systematic reviews and Meta-analysis (PRISMA) guidelines to answer a Population, Intervention, Comparison, and Outcomes (PICO) question: “In patients undergoing orthodontic treatment (P) with clear aligners (I), what are the changes or effects observed in the oral cavity (O) when comparing before and after treatment (C)”.

## 2. Methods

In order to organize the current scientific knowledge on the toxicity of invisible aligners, a systematic search of studies available in the medical literature was carried out in the Pubmed, Scopus, and Cochrane electronic databases to identify relevant articles. This systematic review was conducted following the Preferred Reporting Items for Systematic reviews and Meta-analysis (PRISMA) guidelines to answer a question formulated according to the Population, Intervention, Comparison, and Outcomes (PICO) strategy. This review will also be registered in the OSF database with the registration DOI: https://doi.org/10.17605/OSF.IO/HB2WQ, (accessed on 7 November 2024). The search results were imported into Rayyan [19] to help visualize and operationalize the selection of articles, and the Joanna Briggs Institute (JBI) [20] evaluation tool was used to assess the methodological quality of the studies.

### 2.1. Search Strategy

The PICO question for this review was as follows: in patients undergoing orthodontic treatment (P) with clear aligners (I), what are the changes or effects observed in the oral cavity (O) when comparing before and after treatment (C)?

The following terms were used in the searches: (clear aligners OR Invisalign) AND (biocompatibility OR toxicity OR (biological effects)). For years, Invisalign dominated the clear aligner industry, to the point where its name became synonymous with the product itself. Many patients refer to clear aligners simply as “Invisalign” rather than using the generic term. Research from the past decade suggests that the term "Invisalign" gained widespread popularity because people prefer it over the general name for the treatment. For this, the term “Invisalign” was added in the key search.

The following search equations were used for the different databases, and the following results were obtained:

PubMed/Medline: (clear aligners OR Invisalign) AND (biocompatibility OR toxicity): filters: Human, English, in the last 10 years; retrieved: 367 results.

Cochrane: (clear aligners OR Invisalign) AND (biocompatibility OR toxicity): filters: Human, English, in the last 10 years; retrieved: 15 results.

Scopus database: (clear aligners OR Invisalign) AND (biocompatibility OR toxicity): filters: Human, English, in the last 10 years; retrieved: 31 results.

### 2.2. Sources of Information

A systematic search was carried out on 21 September in the National Institute of Health database. The search was completed on 22 September in the following databases: Pubmed, Cochrane Library, and Scopus.

The articles obtained were imported into bibliographic reference software. The searches were carried out with filters for humans, with text in English and Portuguese, and published between 2014 and 2024.

### 2.3. Eligibility Criteria

To present the relevant results in this review and to address the question, it was necessary to establish well-defined inclusion criteria. In this way, articles dealing with orthodontic treatment with transparent aligners without restricting the age or gender of the participants were included. Therefore, this systematic review was based on aggregating all the information on the toxicity, biocompatibility, and side effects of clear aligners, and the following inclusion criteria were defined for the selection of publications: (1) written in English, (2) published between 2014 and 2024, (3) studies resulting from scientific research on clear aligners, (4) studies describing side effects related to the use of clear aligners, (5) in vitro and in vivo studies, and (6) with full text available.

After the initial assessment, the articles that actually met the inclusion criteria were read in full and those that did not meet at least one of the following exclusion criteria were excluded: (1) case reports, (2) systematic reviews, (3) articles without information on the toxicity and adverse effects of clear aligners, (4) studies that used culture methods as a method of assessing the microbiota, and (5) orthodontic appliances such as retainers, breaker appliances, and expander appliances. The articles selected from the included studies were read in full to ensure that they only dealt with studies on the toxicity of clear aligners and their side effects.

### 2.4. Study Selection

After searching the literature, two independent researchers (PL and MF) filtered the relevant articles that fit the study, analyzing the title and abstract to select the studies. Any disagreements between reviewers were discussed with a third author (AG). Cohen’s Kappa test was used to assess the agreement between the reviewers. The Rayyan Intelligent Systematic Review Platform was used to assist in the systematic review process [19]. Reviewers independently extracted data from the articles selected for analysis. The information collected during data collection was as follows: authors, year of publication, title of the article, study design, type of participants, number of participants, age of participants, type of diagnosis and treatment, period of clinical follow-up, materials and substances used, and the outcome assessed.

### 2.5. Methodological Quality Analysis

The risk of bias and quality of study evaluation was performed by two researchers through the Quality Assessment Tool For In Vitro Studies (QUIN Tool) [21]. This tool includes 12 points for quality assessment of the included studies in the present systematic review. Cohen’s kappa coefficient test was used to evaluate the agreement level between the investigators. 

## 3. Results and Discussion

In September 2024, data were collected, and a systematic literature search was carried out which identified 413 potentially relevant references, 367 of which came from the PubMed/Medline database, 31 from Scopus, and 15 from Cochrane. Nineteen duplicates were found and excluded. Based on the information provided in the title and abstract, 360 were considered ineligible. The main reasons for not including the articles were as follows: (1) unrelated topic; (2) wrong result; (3) review; (4) wrong population; (5) being in humans; (6) wrong study; (7) wrong study duration. Thirty-four articles were analyzed in full to gather more detailed information. Twenty-seven articles were excluded for the following main reasons: (1) wrong result; (2) wrong population; (3) wrong study; (4) no full text available.

**Figure 1:**
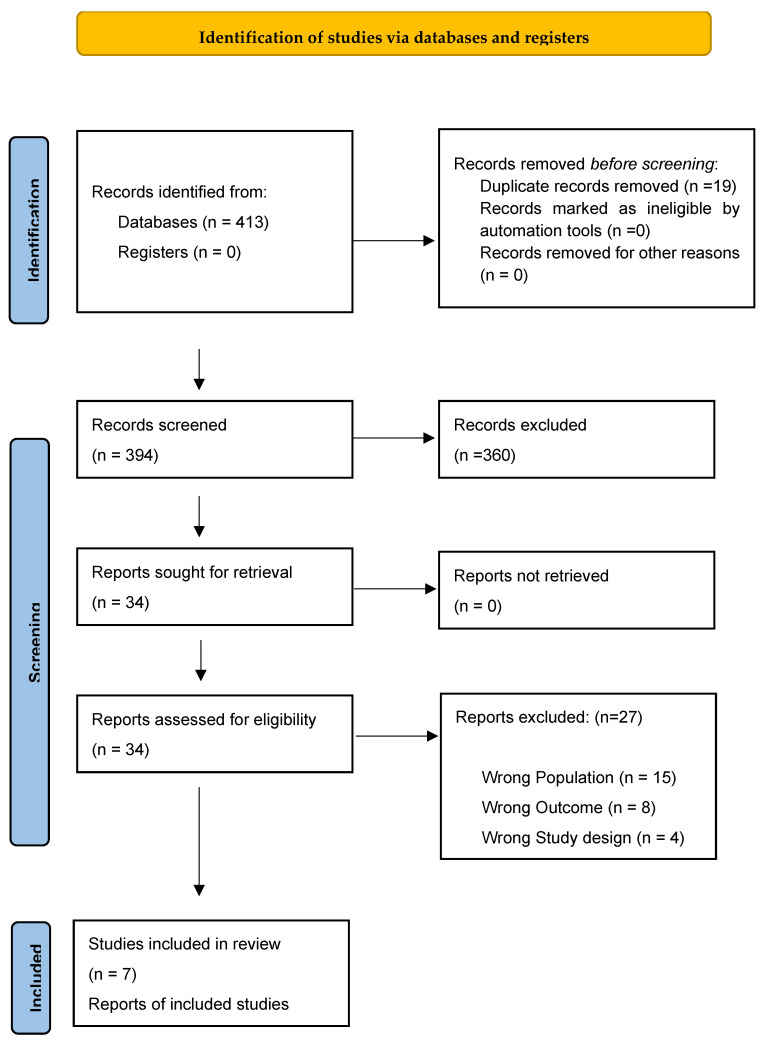
Article selection according to PRISMA guidelines.

Finally, seven articles were included in the following review. The study selection process is shown in Figure 1 and the characteristics of the seven studies included in this systematic review are presented in Table 1.

A total of 413 articles were obtained, 367 of which came from the PubMed/Medline database, 31 from Scopus, and 15 from Cochrane. A total of 19 duplicates were excluded and based on the information provided in the title and abstract, 360 were found to be ineligible. A total of 34 articles were reviewed in full to gather more detailed information and 27 were excluded by applying the inclusion and exclusion criteria. Finally, seven articles were included in this review.

The articles were analyzed by collecting relevant data that was organized on an Excel table (Table 1) by author/year; article title; study design; appliance studied; material; objectives; substances analyzed; and conclusions.

The evaluation of cytotoxicity across various clear aligner materials reveals significant differences in biocompatibility, largely influenced by material composition and processing methods. Among the tested materials, Duran exhibited the highest cell viability (84.6%) and the lowest cytotoxicity, followed closely by SmartTrack (78.8%) and Zendura (74.4%) [7]. Biolon showed the most pronounced cytotoxicity (64.6%), suggesting limited biocompatibility and warranting caution in clinical use [7]. Similarly, Alhendi et al. (2022) [25] reported a dose-dependent reduction in human gingival fibroblast viability for several commercial aligners, including Invisalign and SureSmile, indicating slight to moderate toxicity [25]. Nemec et al. (2023) [22] showed that saliva Invisalign patients did not harm the viability or proliferation of hGFs or oral epithelial. Moreover, Invisalign did not promote an increase in the expression of inflammatory mediators, as occurred in bracket patients [22]. Notably, Dinu et al. (2024) demonstrated that two thermoplastic aligners (CA1 and CA2) maintained over 82% cell viability, confirming their safety according to the EpiDerm™ model standards [1]. Yu et al. (2022) [23] and Yan et al. (2024) [24] also observed low cytotoxicity and preserved cell morphology in aligners composed of thermoplastic polyurethane and fluoride-coated plastic, respectively. Importantly, several studies emphasized that thermoforming processes can significantly increase cytotoxicity, particularly in materials such as TPU and Zendura [26]. These findings highlight the importance of both material selection and processing techniques in ensuring the biocompatibility of clear aligner systems. Nevertheless, of the seven articles analyzed, five concluded that clear aligners are safe for clinical use, while two reported some degree of cytotoxicity.

The studies selected were analyzed regarding the quality of the study according to the (QUIN Tool) [21] and the results of the analysis are presented in Table 2. Almost all aspects of the analysis were fulfilled except for two articles (Yu, X. et al., 2022 [23]; Yan, J. et al., 2024 [24]).

## 4. Discussion

### 4.1. Biocompatibility and Toxicity of Clear Aligners

The use of thermoplastic materials in clear aligners, such as polyurethane and PET-G, has gained widespread acceptance due to their mechanical properties, biocompatibility, and aesthetic appeal [27]. However, concerns persist regarding their potential to release chemical compounds under specific conditions, such as elevated temperatures, acidic environments, and prolonged wear [12,22]. Understanding these materials’ biocompatibility and toxicity is essential for assessing their long-term safety in orthodontic treatments.

Cytotoxicity in clear aligner materials is influenced by a multifactorial interplay involving chemical composition, material degradation, and external influences such as thermoforming and intraoral exposure. Key mechanisms implicated in this toxicity include the leaching of residual monomers or additives, surface degradation, oxidative stress, and inflammatory cellular responses [28,29]. The main mechanisms identified in the literature involve the leaching of residual monomers or additives, the release of degradation by-products from the material’s surface, and the induction of inflammatory responses triggered by the material itself [30,31]. One of the primary concerns is the potential release of BPA and other monomers. BPA, an endocrine disruptor with known estrogenic activity, has been detected in certain thermoplastic dental materials, raising concerns about its cumulative exposure over prolonged treatment durations [2]. Studies indicate that although the levels of BPA released from aligners generally fall within regulatory safety limits, the long-term effects of prolonged exposure remain unclear, particularly for susceptible populations such as adolescents and pregnant women [1,6]. Moreover, alternative bisphenol derivatives such as BPS and BPF, commonly used in BPA-free plastics, may pose similar health risks, necessitating further investigation into their systemic absorption and biological impact [7]. This leaching is exacerbated by thermal and mechanical stresses encountered during thermoforming and oral wear. For instance, Martina et al. (2019) [7] observed increased cytotoxicity post-thermoforming in Duran, Biolon, and Zendura materials, likely due to enhanced release of monomeric compounds. Their results indicated that while these materials are generally safe for clinical use, Biolon exhibited the highest cytotoxic potential, raising concerns about its long-term safety [32]. Lo et al. [26] expanded on these findings by analyzing a range of thermoplastic materials, including polyurethane and PET, and concluded that under the tested conditions, these materials were generally safe for orthodontic treatments, although some variations in cytotoxicity were observed [33]. Also, saliva acidity may modulate cytotoxic effects by enhancing the solubility of harmful leachates, such as BPA and other monomers [29]. Dinu et al. (2024) [1] found a slight decrease in keratinocyte confluence under acidic conditions, indicating that lower pH can amplify material toxicity in the oral environment.

The degradation products can disrupt cellular function, leading to reduced cell viability and alterations in morphology, particularly in gingival fibroblasts and keratinocytes [31]. Several other studies have assessed the cytotoxicity of clear aligners, investigating their interactions with oral tissues, and, in line with that, the cytotoxicity of different clear aligner systems varies depending on the material composition. Alhendi et al. [25] evaluated multiple aligner systems and found that all the tested thermoplastic materials exhibited some degree of cytotoxicity, ranging from slight to moderate [34]. Another characteristic that needs to be considered is the surface texture, roughness, and chemical stability that could significantly influence the biocompatibility of aligner materials. Thermoforming alters the material structure, potentially increasing surface degradation and cytotoxicity (ref). Lo et al. (2024) [26] noticed that TPU materials, especially after thermoforming, showed reduced viability in periodontal ligament cells, suggesting that structural changes induced during processing increase cellular stress or contact toxicity.

Exposure to clear aligner materials can trigger oxidative stress pathways in oral cells, resulting in the production of reactive oxygen species (ROS) and inflammatory cytokines. Chronic low-grade inflammation can compromise epithelial integrity and contribute to oral tissue irritation (ref). Nemec et al. (2023) [22] reported an increased expression of pro-inflammatory mediators (IL-6, IL-8, and MCP-1) in gingival fibroblasts and epithelial cells exposed to aligner-conditioned saliva, indicating an inflammatory cellular response likely tied to material–saliva interactions. Nevertheless, their findings revealed minimal differences in inflammatory responses before and after treatment, suggesting that orthodontic therapy does not significantly alter saliva composition in terms of inflammatory mediators [24].

In addition to cytotoxicity concerns, some research has focused on the potential irritant properties of clear aligners. Dinu et al. [1] conducted in vitro and in vivo biocompatibility studies on two types of clear aligners (CA1 and CA2), assessing their safety profiles. Their findings confirmed that both aligner types did not induce significant cytotoxicity or irritation, supporting their suitability for orthodontic use [35]. Furthermore, Yu et al. [23] explored the mechanical properties of clear aligners and found that polymeric materials exhibited good durability and flexibility when molded onto 3D-printed dental models, which is crucial for maintaining orthodontic forces without compromising safety [36].

Toxic effects appear to be scaled with the concentration of material exposure, pointing to a dose–response relationship, as stated in the study by Alhendi et al. (2022) [25] that demonstrated that increasing concentrations of extract solutions from multiple aligner systems led to greater reductions in fibroblast viability.

In contrast to concerns regarding cytotoxicity, some advancements in aligner materials aim to enhance biocompatibility. Yan et al. [24] evaluated a novel fluoride-coated clear aligner material with antibacterial and enamel remineralization properties. Their study demonstrated that fluoride-coated aligners exhibited antibacterial activity, promoted fluoride recharge, and maintained appropriate physicochemical properties, suggesting a potential avenue for reducing adverse effects associated with prolonged aligner wear [37].

While current evidence suggests that clear aligners are generally safe, variations in material composition, manufacturing processes, and environmental conditions can influence their toxicity profile. Some thermoplastic materials may degrade under high temperatures or acidic conditions, increasing the release of potentially harmful substances. Patients are advised to avoid consuming hot beverages or acidic foods while wearing aligners to minimize these risks [38]. Furthermore, proper aligner maintenance, including regular cleaning with non-abrasive agents, is crucial for preventing bacterial accumulation and ensuring continued safety during orthodontic treatment [38].

Given these findings, continued research is necessary to assess the long-term impact of clear aligner materials on both oral and systemic health. Standardized testing protocols for material toxicity and biocompatibility should be established to ensure the safety of emerging aligner materials. Future studies should also explore patient-specific factors, such as variations in oral pH, saliva composition, and hygiene practices, which may influence the extent of material degradation and chemical leaching [39].

### 4.2. Effects of Aligners on Oral Health

The continuous use of clear aligners, typically for 20–22 h per day, creates a unique oral environment that can influence microbial composition, saliva flow, and overall oral health. Several studies have explored these effects, identifying potential risks such as alterations in the oral microbiome, increased bacterial accumulation, xerostomia, and gingival inflammation [27]. While clear aligners offer improved hygiene compared to fixed appliances, they still pose challenges that require careful patient management and preventive strategies [12].

One of the most significant concerns associated with clear aligner therapy is the alteration in the oral microbiome. The enclosed space created between the aligner and the teeth provides a favorable environment for bacterial proliferation, particularly cariogenic and periodontal pathogens. Nemec et al. [22] investigated the effects of Invisalign aligners on saliva composition and found minimal inflammatory changes before and after treatment, suggesting that aligners do not significantly alter inflammatory responses in saliva [22]. However, their study did not assess bacterial shifts, which remain a critical factor in oral health during treatment.

Other research has highlighted the increase in cariogenic bacteria due to prolonged aligner wear. Alhendi et al. [25] evaluated multiple clear aligner systems and found that thermoplastic materials used in aligners facilitated bacterial adherence, leading to moderate cytotoxic effects on gingival fibroblasts [2]. These findings align with previous reports indicating that aligners may create conditions conducive to biofilm formation, increasing the risk of enamel demineralization and white spot lesions [12].

Similarly, Martina et al. [7] analyzed the cytotoxicity of different aligner materials (Duran, Biolon, and SmartTrack) and found that while they are generally safe, they still pose some risks of bacterial accumulation [6]. Lo et al. [26] expanded on this by assessing polyurethane and PET aligners, concluding that they are generally safe but require strict oral hygiene to prevent bacterial colonization [7].

Beyond microbial changes, xerostomia (dry mouth) is another commonly reported side effect of aligner use. The presence of an aligner covering the dental arches reduces salivary circulation, which can compromise the oral cavity’s natural defense mechanisms. Saliva plays a crucial role in buffering acids, remineralizing enamel, and cleansing bacterial deposits. Dinu et al. [12] confirmed that while certain clear aligner materials showed good biocompatibility, prolonged use can contribute to dryness and discomfort, especially in patients with pre-existing salivary deficiencies [24]. Managing xerostomia involves increasing water intake, using saliva substitutes, and avoiding dehydrating substances such as caffeine and alcohol while wearing aligners [34].

Another effect of aligners on oral health is gingival irritation and mild inflammation. The mechanical irritation caused by the aligners, particularly along the gingival margins, can lead to localized inflammation and soreness. Yan et al. [24] examined a novel fluoride-coated aligner and found that its antibacterial properties reduced plaque accumulation, potentially lowering gingival inflammation risks [32]. This suggests that advancements in aligner materials could mitigate some of the adverse effects currently observed in conventional aligners.

Furthermore, Yu et al. [23] explored the mechanical and chemical stability of aligners molded onto 3D-printed dental models. Their findings demonstrated that well-fitted aligners reduce irritation but emphasized that patient compliance with oral hygiene routines is essential in preventing gingival issues [33].

From a clinical perspective, maintaining optimal oral hygiene while wearing aligners is critical in preventing adverse effects. Patients should be instructed to clean aligners thoroughly using appropriate cleaning agents, avoid sugary and acidic foods that may exacerbate bacterial growth, and ensure consistent oral care practices, including brushing, flossing, and regular dental check-ups [35]. In addition, orthodontists should assess individual patient risks and recommend adjunctive treatments, such as antimicrobial mouth rinses, to minimize bacterial colonization.

Future research should focus on the long-term evaluations of aligner-associated microbiome shifts and the cumulative impact on periodontal and dental health. Additionally, material innovations, such as antimicrobial and fluoride-releasing aligners, may offer new strategies for mitigating oral health concerns associated with prolonged aligner use [36]. Given the growing popularity of clear aligners, further clinical trials are necessary to establish best practices for maintaining oral health during orthodontic treatment.

While clear aligners remain a convenient and effective alternative to fixed appliances, their impact on oral health should not be overlooked. By implementing evidence-based preventive measures and advancing material technologies, the potential risks associated with aligners can be minimized, ensuring that patients achieve both functional and aesthetic benefits without compromising their oral health [37].

### 4.3. Preventive Measures and Alternative Materials

To mitigate the risks associated with clear aligners, including cytotoxicity, microbial accumulation, and material degradation, preventive strategies and material advancements are crucial. Research has focused on improving aligner compositions and clinical recommendations to enhance safety and efficacy.

One of the most promising strategies is integrating antimicrobial and remineralization agents into aligners. Yan et al. [24] demonstrated that fluoride-coated aligners exhibit antibacterial properties and support enamel remineralization, reducing the risk of demineralization during treatment [27]. Antimicrobial coatings, such as silver nanoparticles or chlorhexidine-releasing polymers, have also been explored to limit bacterial growth on aligner surfaces [12].

Patient behavior significantly influences aligner safety. Alhendi et al. [25] noted that thermoplastic materials, while generally safe, exhibit slight to moderate cytotoxicity, which may increase with exposure to high temperatures and acidic environments [22]. Patients should avoid consuming hot or acidic beverages while wearing aligners and adhere to proper hygiene practices, including regular aligner cleaning with non-abrasive agents [2].

Material advancements aim to improve aligner biocompatibility. Dinu et al. [1] found that newer thermoplastics exhibited excellent safety profiles with no significant cytotoxic effects [12]. Lo et al. [26] further assessed the safety of polyurethane- and PET-based aligners, confirming their suitability but emphasizing the need for long-term evaluations [6]. These findings suggest that continued innovation in material science can enhance aligner performance while minimizing potential risks.

Clinically, orthodontists can implement preventive measures such as recommending fluoride mouth rinses and ensuring precise aligner fit to reduce gingival irritation. Martina et al. [7] emphasized the importance of individualized care for patients prone to periodontal issues. Yu et al. [23] further highlighted the role of aligner design in minimizing discomfort and optimizing treatment outcomes [24].

Future research should focus on developing aligners with enhanced antimicrobial properties, improved resistance to degradation, and better biocompatibility. Innovations such as shape-memory polymers may further improve patient experience and long-term treatment success [34]. By integrating advanced materials and preventive strategies, orthodontic treatments can continue to evolve toward safer and more effective solutions.

### 4.4. Clinical Implications and Future Research Directions

The findings of this systematic review underscore the importance of continued research into the long-term safety and biocompatibility of clear aligners. While clear aligners have transformed orthodontic treatment by providing a more aesthetic and convenient alternative to traditional braces, their impact on oral and systemic health remains an area of active investigation. The potential for chemical leaching, alterations in the oral microbiome, and risks of gingival inflammation emphasize the need for further clinical evaluation and material innovation.

One of the key clinical implications of this review is the necessity for orthodontists to consider material composition when selecting aligners for patients. Studies such as those by Alhendi et al. [25] and Martina et al. [7] have shown that while most aligner materials are generally safe, some exhibit moderate cytotoxicity, particularly Biolon and certain PET-based thermoplastics [12,27]. These findings highlight the importance of material selection, particularly for patients with pre-existing sensitivities or periodontal conditions.

Another crucial consideration is the impact of aligners on the oral microbiome. As noted by Nemec et al. [22], aligner use does not significantly alter inflammatory mediators in saliva, yet their enclosed environment facilitates bacterial accumulation [22]. Lo et al. [26] further emphasized that while most materials are biocompatible, they still require strict oral hygiene protocols to prevent microbial imbalances [2]. These insights suggest that orthodontists should emphasize meticulous hygiene practices, possibly incorporating antimicrobial mouth rinses or recommending fluoride-coated aligners like those studied by Yan et al. [24] to mitigate bacterial proliferation [1].

Looking forward to future research should focus on optimizing aligner materials to enhance their safety while maintaining their mechanical properties. Dinu et al. [1] found that certain thermoplastics demonstrated excellent safety profiles with no significant cytotoxic effects, pointing toward the potential for improved materials with enhanced durability and reduced toxicity [6]. Similarly, Yu et al. [23] suggested that advancements in material technology, such as 3D-printed custom aligners, may allow for better adaptation to individual dental structures, reducing mechanical irritation and improving patient compliance [7].

Finally, the regulatory landscape surrounding aligner materials must continue evolving to ensure patient safety. Standardized testing protocols for material toxicity, leaching potential, and long-term biological interactions should be implemented across manufacturers. Future studies should prioritize long-term clinical trials assessing the systemic effects of extended aligner use and cumulative exposure to potential chemical leachates.

By integrating these advancements into clinical practice, orthodontists can optimize treatment outcomes while prioritizing patient safety. Through material innovation, improved hygiene protocols, and comprehensive patient education, clear aligner therapy can continue to offer a safe and effective orthodontic solution. Future research must address the gaps identified in this review to refine aligner technology, ensuring that it remains a biocompatible and sustainable choice for patients worldwide.

In the absence of such developments, it is the dentist’s responsibility to guide patients in implementing preventive measures to ensure the safe use of materials. Keeping aligners clean is crucial to avoid bacterial accumulation and prevent oral health issues. They should be brushed daily using a soft toothbrush and cleaning products specifically designed for aligners. In addition to routine cleaning, patients should take certain precautions to maintain both the aligners’ integrity and their oral health. Aligners must be removed before eating or drinking, as food and beverages can stain or damage the material. Consuming acidic or sugary drinks while wearing aligners can foster bacterial growth and elevate the risk of cavities. If such drinks are consumed, the aligners should be taken out beforehand.

Lastly, patients must adhere to the aligner replacement schedule set by their orthodontist. Natural wear over time can reduce the aligner’s effectiveness if not replaced as directed.

## 5. Conclusions

The widespread use of clear aligners needs a thorough understanding of their biocompatibility, potential toxicity, and long-term impact on oral health. While current evidence suggests that clear aligners are generally safe, concerns remain regarding the chemical leaching of thermoplastic materials, bacterial accumulation due to reduced saliva flow, and mild inflammatory responses. Our review emphasizes that although most materials are biocompatible, some exhibit moderate cytotoxicity, underscoring the need for continued research and improvements in material composition.

## Figures and Tables

**Table 1:** Characteristics of the 7 studies included in this systematic review.

Author/Year	Study Design	Appliance Studied/Materials	Intervention Time	Goals	Substances Analyzed	Outcomes	Conclusions
Nemec, M. et al., 2023 [22]	In vitro/In vivo	Invisalign aligners (SmartTrack)	First 6 months of treatment	Compare the effects of saliva from patients with Invisalign aligners and brackets on human gingival fibroblasts and oral epithelial cells.	Pro-inflammatory mediators; IL-8 production	Increase in the expression of pro-inflammatory mediators (IL-6, IL-8, MCP-1) in the analyzed cells in saliva samples of bracket patients. With the progression of orthodontic treatment (from 0 to 6 months), there was a tendency for a decrease in IL-8 production.	The type of orthodontic treatment (Invisalign vs. brackets) does not significantly influence the saliva’s effect on the inflammatory or barrier properties of oral cells. The changes seen during treatment were minor and transient.
Dinu, S. et al., 2024 [1]	In vitro	Two clear aligner types (Thermoplastic)	24 h	Assess the cytocompatibility and safety profile.	HaCaT keratinocytes and HGF fibroblasts (in vivo) in artificial saliva; chorioallantoic membrane	No relevant changes were observed in the morphology of HaCaT cells, except for a slight decrease in confluence in the more concentrated acidic saliva. Testing on a 3D reconstructed human epidermis (EpiDerm™ model) showed viability above 82% with all samples (the threshold for irritation is <50%).	The results suggest that CA1 and CA2 aligners are suitable materials for use, as they did not present significant cytotoxicity and were not irritating, confirming the safety and compatibility of these materials.
Yu, X. et al., 2022 [23]	In vitro	Angelalign (Thermoplastic polyurethane)	No data	Feasibility of using ’transparent’ polymeric aligners molded into personalized 3D printed dental models.	Poliuretan (polyurethane films were molded via thermoforming into custom 3D printed dental models)	Thermoplastic polyurethane demonstrated excellent biocompatibility: low cytotoxicity, hemolysis <5%, and preserved cell morphology.	The aligner material had good mechanical properties.
Yan, J. et al., 2024 [24]	In vitro	Fluoride-coated clear aligner plastic	26 h	Assess the physicochemical properties and biocompatibility of a fluoride-coated clear aligner plastic.	Human fibroblasts were placed in a Minimum Essential Medium with 10% Fetal Bovine Serum and cell counts were evaluated.	Fluoride-coated clear aligners did not compromise the cell viability of human fibroblasts.	FCAP had antibacterial, fluoride recharge, and enamel remineralization capabilities with appropriate physicochemical properties and biocompatibility.
Martina, S. et al., 2019 [7]	In vitro	Duran, Biolon, Zendura, and SmartTrack aligners	14 days	Assess the in vitro cytotoxicity of different thermoplastic materials for clear aligners on human primary gingival fibroblasts incubated in Dulbecco’s Modified Eagle’s Medium.	Aligners with plastic materials (Duran, Biolon, Zendura, and SmartTrack)	All the materials showed mild cytotoxicity after 14 days of incubation with HGFs. Observed cell viability: Duran: 84.6% ± 4.0 (lowest cytotoxicity); SmartTrack: 78.8% ± 6.3; Zendura: 74.4% ± 2.3; Biolon: 64.6% ± 3.3 (highest cytotoxicity). Thermoforming significantly increased the cytotoxicity of Duran, Biolon, and Zendura materials.	All the materials showed some level of cytotoxicity, with Biolon being the most cytotoxic. Although they are generally safe for clinical use, caution is advised, especially with materials such as Biolon and Zendura.
Alhendi, A. et al., 2022 [25]	In vitro	Invisalign, Eon, SureSmile, and Clarity	30 days	Evaluate and compare the cytotoxicity of multiple clear aligner systems.	HGFs were incubated in a saline solution.	The cell viability of the HGFs decreased with increasing solution concentration, indicating a dose-dependent relationship.	The thermoplastic materials used by all the tested systems presented some degree of toxicity (slight to moderate)
Lo, I. et al., 2024 [26]	In vitro	Duran, Keystone, Zendura, Essix C+, Maxflex, and Leone (Thermoplastic polyurethane and copolyester polyethylene terephthalate)	14 days	Evaluating the cytotoxicity of different clear aligner materials to ensure safety and biocompatibility.	Human periodontal ligament cells (HPDL) were incubated in a Minimum Essential Medium with 10% Fetal Bovine Serum.	The HPDL cells maintained high cell viability after exposure to most of the materials tested, indicating low toxicity of the aligners, especially those composed of PETG (polyethylene terephthalate co-1,4-cyclohexylenedimethylene terephthalate) and PET (polyethylene terephthalate copolyester), especially in their original form, before thermoforming. Some thermoformed TPU (thermoplastic polyurethane) materials showed a more significant reduction in the viability of the HPDL cells.	The results indicate that under the conditions tested, these materials are generally safe for use in orthodontic treatments.

**Table 2:** Results of the analysis of the Quin Tool appraisal checklist for the critical evaluation of the in vitro studies.

Author/Year	A	B	C	D	E	F	G	H	I	J	K	L	Score	Bias Evaluation
Nemec, M. et al., 2023 [22]	2	2	2	2	2	0	NA	2	NA	NA	2	2	16	88.9 (Low Risk)
Dinu, S. et al., 2024 [1]	2	2	2	2	2	0	NA	2	NA	NA	2	2	16	88.9 (Low Risk)
Yu, X. et al., 2022 [23]	2	1	2	1	2	0	NA	2	NA	NA	2	2	14	77.8 (Low Risk)
Yan, J. et al., 2024 [24]	2	2	2	1	2	0	NA	2	NA	NA	2	2	15	83.3 (Low Risk)
Martina, S. et al., 2019 [7]	2	2	2	2	2	0	NA	2	NA	NA	2	2	16	88.9 (Low Risk)
Alhendi, A. et al., 2022 [25]	2	2	2	2	2	0	NA	2	NA	NA	2	2	16	88.9 (Low Risk)
Lo, I. et al., 2024 [26]	2	2	2	2	2	0	NA	2	NA	NA	2	2	16	88.9 (Low Risk)

A: clearly stated aims/objectives; B: detailed explanation of sample size calculation; C: detailed explanation of sampling technique; D: details of comparison group; E: detailed explanation of methodology; F: operator details; G: randomization; H: method of measurement of outcome; I: outcome assessor details; J: blinding; K: statistical analysis; L: presentation of results.

## Data Availability

The raw data supporting the conclusions of this article will be made available by the authors on request.
